# Secular changes in severity of intellectual disability in tuberous sclerosis complex: A reflection of improved identification and treatment of epileptic spasms?

**DOI:** 10.1002/epi4.12111

**Published:** 2018-04-06

**Authors:** Charlotte Tye, Laura E. Thomas, Julian R. Sampson, Julia Lewis, Finbar O'Callaghan, John R. W. Yates, Patrick F. Bolton

**Affiliations:** ^1^ Department of Child & Adolescent Psychiatry and MRC Social Genetic & Developmental Psychiatry Centre Institute of Psychiatry, Psychology & Neuroscience King's College London London United Kingdom; ^2^ Division of Cancer and Genetics Institute of Medical Genetics Cardiff University School of Medicine Cardiff United Kingdom; ^3^ Mental Health and Learning Disabilities Division Anuerin Bevan University Health Board Newport United Kingdom; ^4^ Institute of Child Health University College London London United Kingdom; ^5^ Department of Medical Genetics University of Cambridge Cambridge United Kingdom

**Keywords:** Epilepsy, Infantile spasms, IQ, Tuberous sclerosis complex, Vigabatrin

## Abstract

Tuberous sclerosis complex (TSC) is a multisystem genetic disorder caused by mutations in *TSC1* or *TSC2*. Epilepsy occurs in 80%‐90% of affected individuals during their lifetime, and up to one‐third of children with TSC will develop epileptic (infantile) spasms, for which vigabatrin has been shown to be particularly effective. Epilepsy severity and epileptic spasms are consistent markers of risk for the development of intellectual impairment in TSC. Although previous studies demonstrate a bimodal distribution of intellectual ability in TSC, recent findings suggest a unimodal distribution, which may reflect a change in IQ distribution over time. We compared 3 large historical UK cohorts of TSC (n = 331) that show varied distributions of intellectual ability, first ruling out differences in study methodology. Later‐born individuals had a higher frequency of reported spasms and higher likelihood of vigabatrin administration, but were less likely to have profound intellectual impairment, compared to the earlier‐born individuals. Our findings suggest that epileptic spasms went undetected in the older patients and therefore were not treated, leading to a higher occurrence of profound impairment, whereas the later born cohort had better access to treatment. These findings support the importance of early identification and treatment of seizures in TSC.

Tuberous sclerosis complex (TSC) is a rare genetic disorder caused by a mutation of *TSC1* or *TSC2* and characterized by the multisystemic growth of tumor‐like lesions called hamartomas. Cortical tubers and peritubular cortex act as epileptogenic foci during the early postnatal period and are associated with an increased risk of epilepsy, intellectual impairment, and behavioral disturbances. Approximately 90% of individuals with TSC will develop epilepsy, with seizures usually beginning in the first year of life.^1^ Epileptic (infantile) spasms are observed in around half of all cases, associated with developmental regression and intractable epilepsy.^2^

Approximately 50% of individuals develop an intellectual disability, which can range from mild to profound.^3^ Epilepsy severity and epileptic spasms are a consistent marker of risk for the development of intellectual impairment in TSC. Prospective longitudinal studies indicate poor intellectual development in the first 2 years of life in infants with spasms,^4^ and a clear impairment in the development of IQ after the onset of spasms has been demonstrated compared to nonspasm seizures.^5^ The risk of epileptic spasms is correlated with the number of cortical tubers and the presence of *TSC2* versus *TSC1* mutations, and contributes to increased epilepsy severity. In line with this, recent findings suggest a cascading risk pathway from type of genetic mutation, to cortical tuber “load,” to epilepsy severity, through to intellectual ability.^6^

Several studies demonstrate a bimodal distribution of intellectual ability in TSC. This indicates that most individuals with TSC fall either into the normal ability range or profoundly impaired range of intellectual ability, and therefore that distinct subgroups may exist, for example, based on type of genetic mutation.^7^, ^8^, ^9^ Recent findings, however, suggest a change to a unimodal distribution in the United Kingdom.^6^ The reason for the change in distribution is unclear and could reflect methodological differences between cohorts, a decline in IQ in a subset of cases reflecting developmental change, or a true change in IQ distribution over time. We sought to address this question by directly comparing the characteristics of 3 large independent cohorts of individuals with TSC.

## Methods

We directly compare and combine findings from 3 cohorts of TSC:

1. The Wessex cohort (n = 108, date of birth [DoB] 1922–1995, age = 4.0–75.0 years, M:F = 1:0.9) was ascertained through a 1998 census in the Wessex area of SW England.^10^ The average reported age at seizure onset was 28.6 months and 47% of the sample had a history of epileptic spasms. Data were not available for the proportion that received vigabatrin treatment. Assessment of intellectual ability was conducted using the Wechsler Adult Intelligence Scale (WAIS‐R), Wechsler Intelligence Scale for Children (WISC‐III), Raven's Coloured Progressive Matrices, or Vineland Adaptive Behavior Scale. The median IQ was 75. Estimated IQ was bimodally distributed, with a skewness of −0.14 and a kurtosis of −1.51; 55.5% had IQ in the normal range, 14% had mild to severe impairments, and 30.5% had profound disability.
2. The Cardiff cohort (n = 98; DoB = 1930–1995, age = 6.0–70.0 years, M:F = 1:0.9), ascertained for molecular genetic research through adult and pediatric medical clinics, regional genetics services, and learning disability services.^8^ The average reported age of seizure onset was 43.3 months and 44% of the sample had a history of epileptic spasms, with 20% of the sample administered vigabatrin. Assessment of intellectual level was conducted using the WAIS‐R, WISC‐III, Raven's Coloured Progressive matrices, or Vineland Adaptive Behavior Scale. The median IQ was 65. Estimated IQ was bimodally distributed with a skewness of 0.09 and a kurtosis of −1.63; 47.5% had IQ in the normal range, 20.5% had mild to severe impairments, and 32% had profound disability.
3. The TS 2000 cohort (n = 125, DoB = 1986–2005, age = 0.3–21.2 years, M:F = 1:0.9), a prospective longitudinal study of children in the United Kingdom aged 0‐16 years newly diagnosed with TSC between January 2001 and December 2005.^6^, ^11^ The average age at seizure onset was 13.5 months and 49% of the sample had a history of epileptic spasms, with 51% of the sample being administered vigabatrin. Assessment of intellectual level was conducted using the Mullen Scales of Infant Development and the Vineland Adaptive Behavior Scales. The median IQ was 64. Estimated IQ was unimodally distributed, with a skewness of 0.83 and a kurtosis of 0.54; 34.5% had IQ in the normal range, 61.5% had mild to severe impairments, and 1% had profound disability.

## Findings

Although there were some differences in the modes of ascertainment between the 3 studies (eg, the TS 2000 Study recruited all newly diagnosed children with TSC over a set period of time, whereas the Wessex sample included all cases known to Wessex services, as well as secondarily ascertained cases), these differences cannot explain the differences observed in IQ distribution across cohorts. In addition, comparable standardized approaches to estimating IQ were utilized. Moreover, there were no differences in the sex ratio (χ^2^ = 0.01, p = 0.99), history of epileptic spasms (χ^2^ = 0.34, p = 0.84), or age at seizure onset (H (2) = 2.02, p = 0.37) by cohort. When considered as a whole, there was no significant difference in IQ between studies (F (1, 324) = 1.98, p = 0.14). When considering rates of intellectual disability (normal >70; mild to moderate 35–70; severe to profound <20–34), significantly fewer individuals in the TS 2000 cohort had severe to profound disability and more had mild to moderate disability, compared to the Wessex and Cardiff cohorts (χ^2^ = 89.91, p < 0.001). Within the subset of individuals with severe to profound disability, there was no significant difference across cohorts by sex, reported age of seizure onset, history of spasms, or type of mutation (*TSC1* versus *TSC2*; all p > 0.05).

A regression of IQ on date of birth (beta = −0.03, p = 0.60) and age (beta = 0.04, p = 0.44), revealed that neither was a significant predictor of IQ. The cohort to which the participant belonged did not significantly predict IQ on its own (beta = −0.07, p = 0.22) or when entered with age (beta = −0.06, p = 0.26) and date of birth (beta = −0.07, p = 0.23), suggesting that differences in IQ are not the result of cohort effects.

### Distribution of genetic mutations across cohorts

Differences in the distribution of IQ may reflect differences in the type and location of the genetic mutation across the cohorts. Germline mutations were known for 94 participants from TS 2000 (75 patients had *TSC2* mutations and 19 had *TSC1*) and 83 from the Cardiff cohort (70 with *TSC2* mutations and 13 with *TSC1)*. Germline mutations were unavailable from the Wessex cohort. There was no difference in the frequency of *TSC1* versus *TSC2* mutations between the London and Cardiff cohorts (χ^2^ = 0.47, p = 0.49). There was no difference in the rate of *TSC1* or *TSC2* missense mutations, mutations in the *TSC1* tuberin interaction domain, mutations in the *TSC2* hamartin binding domain, *TSC2* mutations in the GAP domain, protein truncating *TSC2* mutations, or proximal protein truncating *TSC2* mutations (see Supplementary Materials, Table S1).

### Changes in epilepsy management

**Figure 1:**
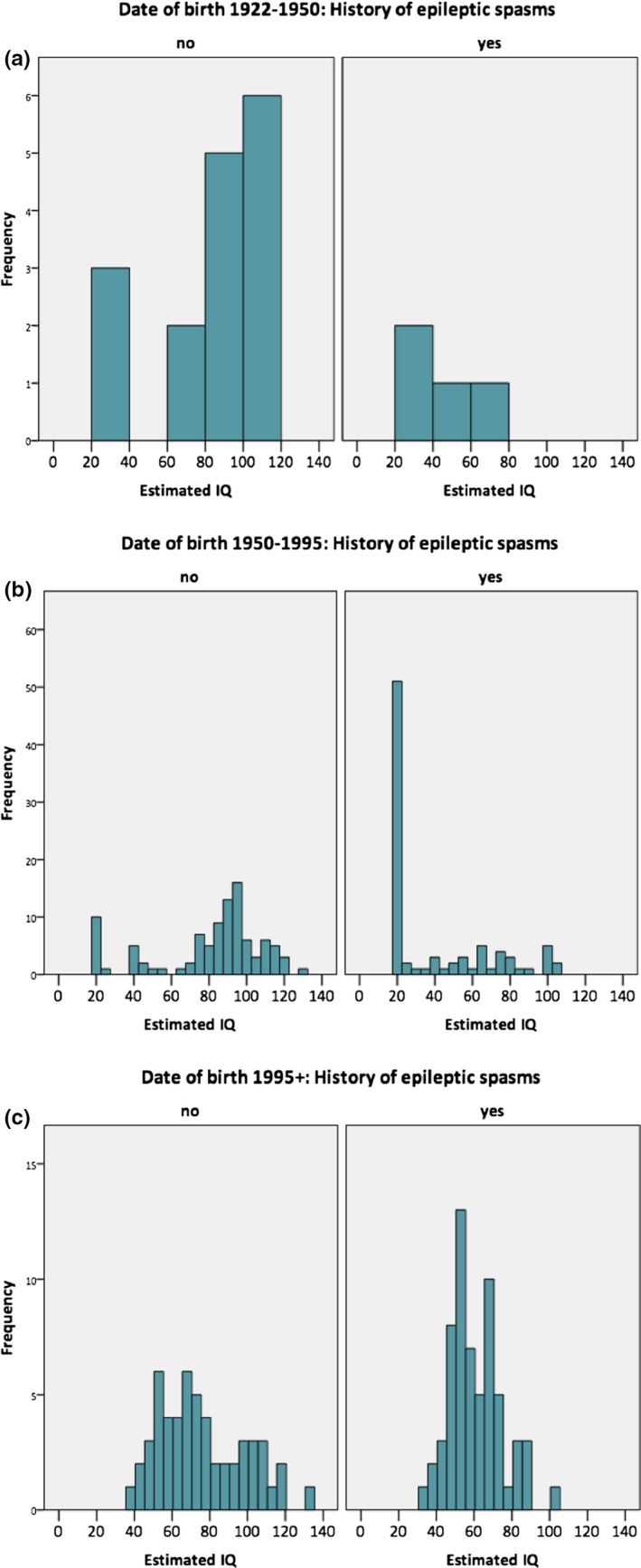
Distribution of intellectual ability by history of epileptic spasms and year of birth across all cohorts: (**A**) 1922–1950; (**B**) 1950–1995, introduction of corticosteroids; (**C**) 1995 onward, introduction of vigabatrin.

An alternative explanation for the change in IQ distribution is improvement in the management of epilepsy, and, in particular, epileptic spasms, in the last 2 decades. The primary approved treatments for children with epileptic spasms are steroid therapy and vigabatrin. Corticosteroids were introduced in the late 1940s, whereas vigabatrin was approved in 1995. In line with this, compared to the Cardiff cohort, significantly more individuals in the TS 2000 cohort received vigabatrin treatment (χ^2^ = 22.67, p < 0.001). To explore this question further, we combined data from all 3 cohorts and stratified by date of birth (before 1950, 1950‐1995, and after 1995). There was a higher rate of vigabatrin treatment (χ^2^ = 23.72, p < 0.001; Cardiff and TS 2000) and history of epileptic spasms in those born after 1995 (χ^2^ = 7.72, p = 0.02; all 3 cohorts; Fig. 1).

If epileptic spasms are a risk factor for intellectual impairment, and their effective treatment leads to less severe disability, we might expect there to be a difference between cohorts for those that have epileptic spasms only, as the TS 2000 cohort had better access to treatment. Consistent with this, there were significant differences in IQ by cohort (F (2,150) = 14.76, p < 0.001) for these individuals; those in the TS 2000 study had significantly higher mean IQ (61) compared to those in the Wessex (39; p < 0.001) and Cardiff (42; p < 0.001) cohorts.

To exclude the possibility that these differences reflected differences in the age of participants at assessment, we matched the individuals from the Wessex and Cardiff samples by age to the TS 2000 cohort (range 25 years, n = 90). Significant differences in IQ by cohort in individuals with a history of spasms were retained between TS 2000 (59), Wessex (43, p = 0.02), and Cardiff (45, p = 0.04).

## Discussion

The findings of the current study suggest that although later‐born individuals with TSC had a higher reported frequency of epileptic spasms, this group was less likely to have profound intellectual impairment. In contrast, those born earlier were less likely to have epileptic spasms recorded, more likely to have profound intellectual impairment, and less likely to have been administered vigabatrin. These observations are consistent with a model in which epileptic spasms went undetected or were detected late in the older patients and therefore were not treated promptly and/or effectively, leading to a higher occurrence of profound impairment (and therefore a bimodal distribution of IQ in TSC).

This hypothesis must be tested directly. For example, it can be postulated that in countries where vigabatrin is not yet available, a bimodal distribution would still be observed. In addition, the possibility that there may be intellectual decline later in development cannot be ruled out. Declines in IQ have been reported in subsets of infants^12^ and children with TSC,^13^ but intellectual ability appears to be stable in most individuals after early childhood. Future analyses of longitudinal cohorts into adulthood are important to reveal whether such subgroups emerge with development and what processes are related to these changes.

Although detection and treatment of epileptic spasms may be important in determining risk for severe to profound intellectual impairment, other risk factors should not be overlooked. History of epileptic spasms is consistently linked to lower intelligence in univariate analyses, yet multifactorial analyses that are able to test the joint effects of several influencing factors have suggested that age at seizure onset is the sole variable for independently predicting intellectual outcome.^4^, ^14^, ^15^ Thus other types of epilepsy, including focal seizures, occurring early in life necessitate prompt management.^16^, ^17^ It is likely that intellectual development in TSC is determined by a complex and dynamic interaction between several risk factors, including number, location, and relative proportion of cortical tubers and type of genetic mutation, in addition to certain features indexing epilepsy severity.^6^, ^14^

These findings have implications for the identification and prevention of the epilepsy‐related risk factors that are involved in shaping intellectual development in TSC. This proposition is timely given recent advances in our understanding of the pathophysiology and treatment of early onset epilepsy in TSC. Clinical seizures are preceded by abnormalities on electroencephalography (EEG) recordings, and preventative treatment of epileptiform discharges may alter the evolution to clinically evident subtle partial seizures and spasms, which may be difficult for parents to identify.^18^, ^19^ For example, infants treated with vigabatrin based on identification of paroxysmal activity on EEG before the onset of clinical seizures had better cognitive outcome at 24 months of age, compared to those treated postseizure onset,^20^ work that is being continued within the EPISTOP project (http://www.epistop.eu). In addition, patients diagnosed with TSC preseizure onset have a lower prevalence of refractory seizures and severe developmental disability, compared to those diagnosed postseizure onset, despite no regular surveillance EEG.^21^

Several clinical trials are ongoing that are focused on neurocognitive outcome in TSC based on identified risk factors, for example, using mammalian target of rapamycin (mTOR) inhibitors that target the downstream effect of the primary mutation (ClinicalTrials.gov Identifier: NCT01289912), through to parent‐based intervention (NCT03422367), and identification of EEG biomarkers of early versus delayed vigabatrin treatment in infants with TSC (NCT02849457). The results of these large‐scale longitudinal and interventional studies of TSC are eagerly awaited. Future work that systematically measures the separate and combined effects of candidate risk factors in neurocognitive development are required to guide routine surveillance and treatment strategies and to improve longer‐term outcome in TSC.

## Disclosure

None of the authors has any conflict of interest to disclose. We confirm that we have read the Journal's position on issues involved in ethical publication and affirm that this report is consistent with those guidelines.

## Supporting information


**Table S1.** Cohort comparisons of mutation frequency by type and domain in both *TSC1* and *TSC2*. Domains are highlighted in gray.
