# Supplementary material for: Secular changes in severity of intellectual disability in tuberous sclerosis complex: A reflection of improved identification and treatment of epileptic spasms?
Source: Epilepsia Open. 2018 Apr 6;3(2):276–80. doi: 10.1002/epi4.12111 (PMC5983114; doi:10.1002/epi4.12111)
Supplement: Supplementary file 1 — Table S1. Cohort comparisons of mutation frequency by type and domain in both TSC1 and TSC2. Domains are highlighted in gray. [file EPI4-3-276-s001.docx]

**Supplementary materials: Secular Changes in Severity of Intellectual Disability in Tuberous Sclerosis Complex: A Reflection of Improved Identification and Treatment of Epileptic Spasms?**

Table S1: Cohort comparisons of mutation frequency by type and domain in both *TSC1* and *TSC2*. Domains are highlighted in grey. Partial gene deletion is denoted as anything less than a whole gene deletion but greater than deletion of half of the gene. Exonic deletion is denoted as deletion of between 1 exon and half of the gene. Proximal protein truncating is denoted as any mutation occurring within the first 22 exons of *TSC2*. TID = Tuberin interaction domain. HID = Hamartin interaction domain. GAP = GTPase activating protein.

| **Gene** | **Mutation Type/Domain** | **Frequency** | | | |
| --- | --- | --- | --- | --- | --- |
|  |  | **TS 2000** | | **Cardiff** | |
|  |  | **Count** | **Percentage** | **Count** | **Percentage** |
| *TSC1* | Whole gene deletion | 1 | 5.3 | 0 | 0.0 |
|  | Partial gene deletion | 0 | 0.0 | 0 | 0.0 |
|  | Exonic deletion | 1 | 5.3 | 0 | 0.0 |
|  | Frameshift deletion | 6 | 31.6 | 7 | 53.8 |
|  | Frameshift insertion | 4 | 21.1 | 2 | 15.4 |
|  | In frame deletion | 0 | 0.0 | 0 | 0.0 |
|  | In frame Insertion | 0 | 0.0 | 0 | 0.0 |
|  | Nonsense | 4 | 21.1 | 4 | 30.8 |
|  | Missense | 1 | 5.3 | 0 | 0.0 |
|  | Splice | 2 | 10.5 | 0 | 0.0 |
|  | Gross Rearrangement | 0 | 0.0 | 0 | 0.0 |
|  | TID | 5 | 26.3 | 1 | 7.7 |
| Total *TSC1* |  | 19 | 20.2 | 13 | 15.7 |
| *TSC2* | Whole gene deletion | 1 | 1.3 | 4 | 5.7 |
|  | Partial gene deletion | 2 | 2.7 | 0 | 0.0 |
|  | Exonic deletion | 6 | 8.0 | 0 | 0.0 |
|  | Frameshift deletion | 10 | 13.3 | 11 | 15.7 |
|  | Frameshift insertion | 2 | 2.7 | 4 | 5.7 |
|  | In frame deletion | 3 | 4.0 | 4 | 5.7 |
|  | In frame insertion | 1 | 1.3 | 0 | 0.0 |
|  | Nonsense | 19 | 25.3 | 17 | 24.3 |
|  | Missense | 13 | 17.3 | 13 | 18.6 |
|  | Splice | 18 | 24.0 | 5 | 7.1 |
|  | Gross Rearrangement | 0 | 0.0 | 12 | 17.1 |
|  | HID | 17 | 22.7 | 8 | 11.4 |
|  | GAP Domain | 19 | 25.3 | 14 | 20.0 |
|  | Proximal protein truncating | 38 | 50.7 | 28 | 40.0 |
| Total *TSC2* |  | 75 | 79.8 | 70 | 84.3 |
| Total number of mutations |  | 94 | | 83 | |
